# B cell tolerance and autoimmunity: Lessons from repertoires

**DOI:** 10.1084/jem.20231314

**Published:** 2024-08-02

**Authors:** Jacques Deguine, Ramnik J. Xavier

**Affiliations:** 1 Immunology Program, Broad Institute of Massachusetts Institute of Technology and Harvard, Cambridge, MA, USA; 2 Center for Computational and Integrative Biology, Massachusetts General Hospital and Harvard Medical School, Boston, MA, USA; 3 Department of Molecular Biology, Massachusetts General Hospital, Boston, MA, USA

## Abstract

Adaptive immune cell function is regulated by a highly diverse receptor recombined from variable germline-encoded segments that can recognize an almost unlimited array of epitopes. While this diversity enables the recognition of any pathogen, it also poses a risk of self-recognition, leading to autoimmunity. Many layers of regulation are present during both the generation and activation of B cells to prevent this phenomenon, although they are evidently imperfect. In recent years, our ability to analyze immune repertoires at scale has drastically increased, both through advances in sequencing and single-cell analyses. Here, we review the current knowledge on B cell repertoire analyses, focusing on their implication for autoimmunity. These studies demonstrate that a failure of tolerance occurs at multiple independent checkpoints in different autoimmune contexts, particularly during B cell maturation, plasmablast differentiation, and within germinal centers. These failures are marked by distinct repertoire features that may be used to identify disease- or patient-specific therapeutic approaches.

## Teaser

We review how technological advances have expanded our ability to map immune receptor repertoires across B cell states in health and disease, and how these datasets reveal distinct processes involved in the generation of autoantibodies.

## Introduction

The ability of adaptive immune responses to recognize antigens depends on a repertoire of receptors generated from the recombination of a set of genetically encoded sequences known as variable, diversity, and joining (V, D, and J) segments at both the heavy and light chain loci. These segments are joined by additional random nucleotides to generate a broad array of receptor chains during B and T cell development. As this process can generate auto-immune responses, these repertoires are then pruned to eliminate highly autoreactive receptors in the naïve adaptive immune cell pool, a phenomenon referred to as central tolerance. Ideally, repertoire maturation will generate a broad naïve repertoire that can potentially recognize a vast range of foreign epitopes and has minimal reactivity to self-antigens, although, as we will discuss further below, it is evident that reactivity to self is not completely eliminated, potentially because a high stringency would limit the breadth of potential responses.

The retained naïve cells are then exported to the periphery where they circulate between secondary lymphoid tissues such as the spleen and lymph nodes. Upon encountering an antigen that binds to their receptor in the appropriate settings, naïve follicular B cells will be activated and expanded, contributing both to the generation of short-lived antibody-producing plasma cells and germinal centers (GCs) where B cells can further expand and undergo somatic hypermutation of their receptor. Somatic hypermutation is essential to select high-affinity clones through competition for antigen binding and T cell help within the GC, further increasing antibody specificity but also potentially leading to the emergence of undesirable specificities and self-reactivity. Ultimately, the GC reaction leads to the generation of both memory B cells and long-lived plasma cells, and the latter continues to secrete antibodies over years to decades, contributing to protection from re-infection and the efficiency of most vaccinal strategies.

The repertoire of an individual is therefore shaped by a complex combination of genetic (the V, D, and J segments themselves as well as variation in the signaling pathways that control B cell development and activation) and environmental factors driven by successive antigen exposures. Our ability to understand both the B cell and antibody repertoires has drastically expanded with the development of next-generation sequencing and single-cell genomics. Here, we will discuss how these methodologies and the findings derived from their application to patient cohorts shed new light on the contribution of B cells and antibodies to human disease, with a particular focus on autoimmunity.

## Structure and genetic variation of the Ig loci

**Figure 1.**
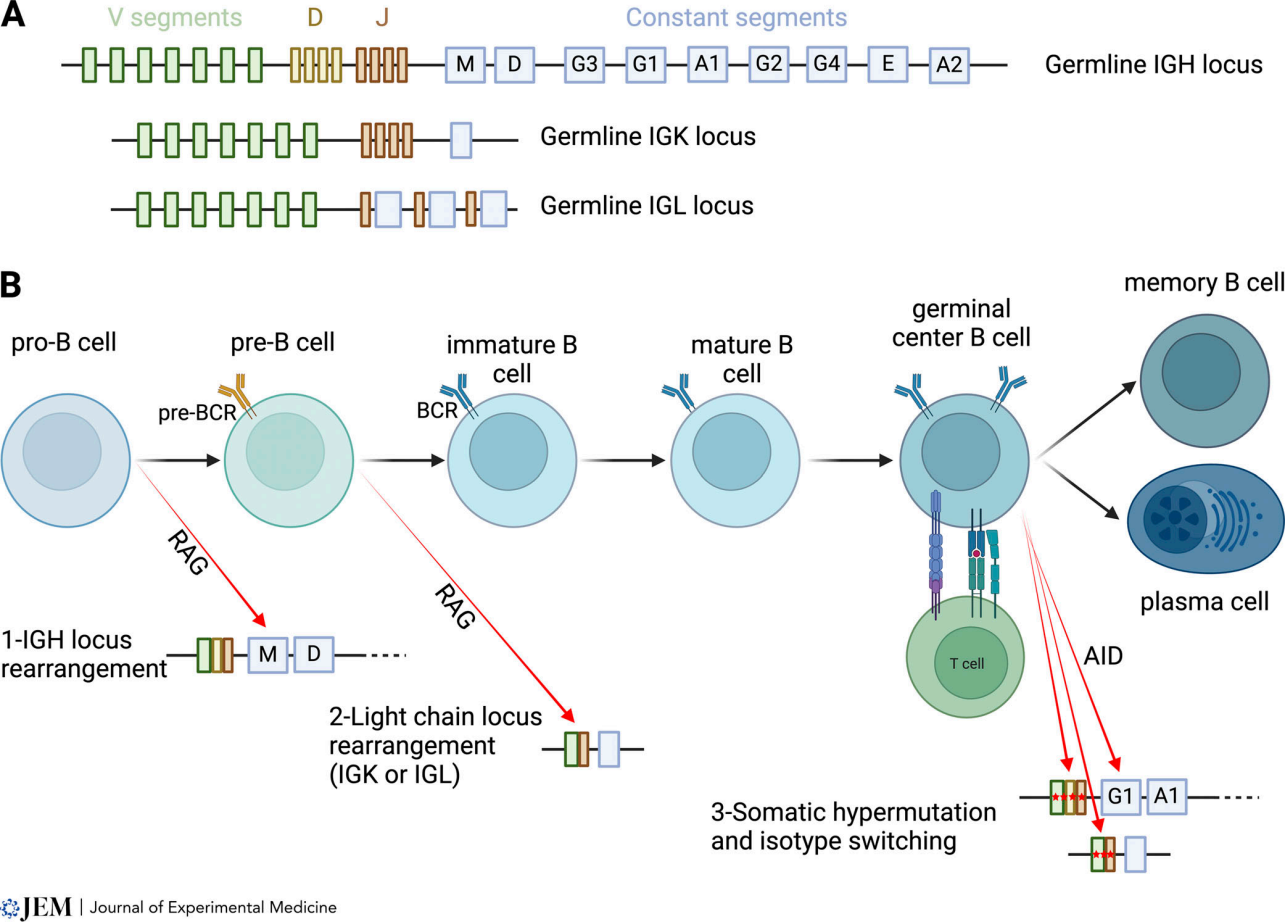
**Rearrangement of germline Ig loci across the lifecycle of B cells. (A)** Schematic of the germline configuration of the IGH, IGK, and IGL loci. IGH contains V, D, and J segments followed by the constant segments, here represented by the letter associated with the Ig isotype, e.g., M for the IgM-associated constant chain Cμ. IGK and IGL only contain V and J segments, followed by a single constant segment for IGK, while in IGL each J segment is associated with its own constant segment. **(B)** Overview of B cell development and activation with key modifications of the IG loci. At the pro-B cell stage, developing B cells rearrange the IGH locus to yield a functional heavy chain and cells that successfully express a pre-BCR pass a first checkpoint to the pre-BCR stage. At this stage, the light chain loci are rearranged to yield a functional light chain. Finally, during or around the GC reaction, AID can lead to somatic hypermutation (red stars in the VDJ/VJ regions represent mutations) or to class-switching among IGH constant segments (here, depicting a locus that has switched to IgG1, where downstream segments including IgA remain available). Created with BioRender.com.

B cell receptors (BCRs) and antibodies are assembled from a heavy chain, encoded by the Ig heavy (IGH) locus, and a light chain that can be derived from either the Ig κ or λ loci (IGK, IGL). These core loci are located on chromosomes 14, 2, and 22, respectively, and are among the most polymorphic across the human genome (Fig. 1 A). Multiple Ig genes are also present as orphons outside of these loci, and there is recent evidence that even non-Ig genes can be recombined interchromosomally to expand the diversity of antibodies in the case of LAIR1-domain-containing antibodies (Tan et al., 2016), but these processes remain poorly characterized and will not be discussed at length here.

The IGH locus is the first to recombine during B cell development to generate the heavy chain from the assembly of V, D, and J segments. The ImMunoGeneTics database (Giudicelli et al., 2005) records 57 functional V, 23 D, and 6 J segments. These segments are highly polymorphic, with, for example, 343 alleles of variable segments, and it is likely that a lot of the variation is still uncharacterized (Watson et al., 2017). Indeed, many studies of diverse populations are uncovering novel alleles at high rates (Calonga-Solis et al., 2019; Khatri et al., 2021). The IGH locus also contains an array of constant heavy chain segments that encode functionally distinct antibody isotypes and are also variable across the human population. The κ light chain locus, IGK, harbors 41 V segments and 5 J segments clustered next to a single C segment. The λ light chain locus, IGL, harbors 33 V segments and differs from IGK in that each of the five J segments is associated with its own C segment. IGL also contains VPREB1, which together with λ5 forms the surrogate light chain. In each locus, adapted recombination signal sequences of diverse strength and chromatin structures appear to maximize a broadly diverse utilization of these sequences (Zhang et al., 2024).

While the stochastic assembly of BCRs from the rearrangements of these loci generates a high diversity of potential sequences within each individual, it is also evident that genetic diversity across the locus can contribute to health and disease. Long-read sequencing approaches demonstrate a striking extent of structural variation, with over half of the IGHV segments being deleted in at least one individual out of a cohort of 154 subjects (Rodriguez et al., 2023). This study also demonstrated the existence of many gene usage quantitative trait loci, which bias the use of specific V segments in the overall B cell population and will be important to consider for repertoire-level analysis. Random nucleotides and later hypermutation can likely generate an almost unlimited set of specificities, yet multiple lines of evidence suggest that germline V segments can favor specific responses, for example, the Phe54 allele of IGHV1-69 promotes the emergence of broadly neutralizing flu antibodies, is differentially distributed across populations, and also affects gene usage (Avnir et al., 2016; Lingwood et al., 2012; Pappas et al., 2014). Similarly, specific IGHV segments are associated with broadly neutralizing HIV antibodies (Kwong and Mascola, 2012; Scheid et al., 2011; West et al., 2012), and approaches that aim to elicit VRC01 class antibodies, linked to IGHV1-2, have shown that allelic variation impacts the ability to elicit these precursors from the naïve repertoire (deCamp et al., 2024; Leggat et al., 2022).

From these observations, it seems plausible that germline variation could favor the emergence of pathogenic autoantibodies, just like it favors the emergence of specific classes of protective antibodies. IGH haplotypes could also underlie some of the repertoire biases observed in autoimmune diseases and discussed hereafter. Indeed, some associations between IGH polymorphisms and disease risk have been reported (Avnir et al., 2016; Parks et al., 2017), but the complex structure of the locus has generally limited the ability of standard genome-wide association study approaches to identify associations. The incorporation of targeted long-read sequencing and tailored computational approaches (Rodriguez et al., 2020) represents an important avenue to identify novel genetic risk factors for the development of autoantibodies, but also to identify association between disease and the functional roles of antibodies encoded by the constant chain, which will be discussed in a separate section.

## Assembly of the BCR and antibody structure

During B cell development (Hardy and Hayakawa, 2001), the heavy chain is assembled first through a D→J then V→DJ joining by the action of the recombination activating genes (*RAG1/RAG2*) (Fig. 1 B). At both junctions, the template-independent polymerase TdT (*DNTT*) will add two to five random nucleotides before the DNA is repaired by non-homologous end-joining. The recombined heavy chain will be expressed alongside the surrogate light chain as a pre-BCR, and successful signaling from this receptor will provide a first checkpoint (Melchers, 2015) for developing B cells. This V→DJ recombination occurs under strict allelic exclusion (Vettermann and Schlissel, 2010), meaning that mature B cells will express a single functional heavy chain. B cells with a productive IGH rearrangement will transiently proliferate and downregulate RAG before initiating recombination at the light chain loci and undergoing selection. Recombination at the light chain loci uses the same machinery but occurs in a single V→J step, initially at the κ locus. In the absence of a productive rearrangement, or if an autoreactive antibody is generated, further rearrangements can occur at the κ and ultimately λ loci.

In mature B cells, an antibody is formed by two heavy chains and two light chains linked by disulfide bonds. The antigen-specificity of the heavy and light chains primarily derived from V(D)J encoded amino acids across the complementarity-determining regions (CDR) 1, 2, and 3. CDR1 and -2 are encoded by the V segment, while CDR3 is located at the V(D)J junction and is maximally variable as it is partially encoded by random nucleotides added by TdT during recombination. The heavy constant region is conserved across naïve B cells at this stage, with the segments encoding for IgM and IgD located proximally to the recombined VDJ segment, enabling B cells to produce both isotypes through alternative splicing. The BCR itself is a membrane-bound complex formed by an antibody dimer (two heavy and two light chains) linked to one heterodimer of Igα and Igβ (also known as CD79A/B) that acts as the signaling unit of the complex. Structural studies of the complex in humans (Ma et al., 2022; Su et al., 2022) and mice (Dong et al., 2022) have shown that BCR assembly is conserved and involves a conserved four-helix bundle of the transmembrane domains of these four chains. However, this structural information also shows that variations in the constant region across isotypes may modify interactions across the extracellular domains and modulate the resulting signaling.

Once the complete BCR is formed, developing B cells pass through tolerance checkpoints that will be discussed at length below given their relevance to autoimmunity. However, it is important to note here that the IG loci can be further edited by the action of the enzyme activation-induced cytidine deaminase (*AICDA*, also known as AID) during activation and the GC reaction (Victora and Nussenzweig, 2022). Specifically in GCs, somatic hypermutation focused on the CDRs enables the mutation of residues potentially involved in antigen binding and the selection of higher-affinity antibodies. AID is also responsible for class-switching to another isotype, although there is evidence that the switch occurs outside of GCs (Roco et al., 2019). In this process, constant regions encoding for IgM/IgD are excised through double-strand breaks and a new constant segment becomes proximal to the recombined VDJ region (Stavnezer et al., 2008). Importantly, because of the order of the constant regions in the germline *IGH* locus, sequential class-switching can occur to more distal regions in this order: *IGHM/IGHD* → *IGHG3* → *IGHG1* → *IGHA1* → *IGHG2* → *IGHG4* → *IGHE* → *IGHA2*. This process can lead to the functional evolution of an antibody clone over time, and studies have suggested that IgG1 precursors are essential for the emergence of high-affinity IgE in allergy (Xiong et al., 2012). A survey of the repertoire suggests that many if not all of these possible transitions can be found in human B cells (Horns et al., 2016), although some pathways may be enriched, such as the development of B cells expressing IgG4 from other IgGs rather than directly from IgM, as seen after repeated mRNA vaccination (Irrgang et al., 2023).

## Methodological approaches to study Ig repertoires

**Figure 2.**
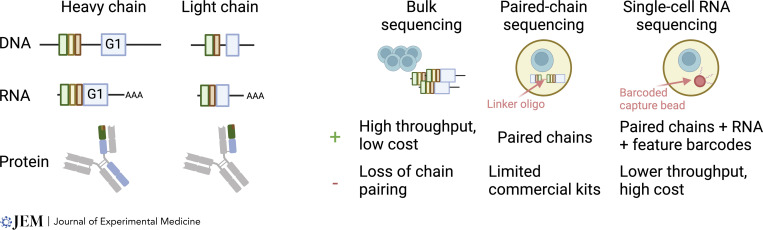
**Antibody characterization through sequencing approaches.** Left: Schematic of the locus (DNA), transcript (RNA with poly-A tail represented by AAA), and resulting antibody (protein) for the heavy and light chain of a representative IgG1 antibody. Constant chain regions are depicted in blue, while antibody-binding fragments are derived from V, D, and J segments (green, yellow, and red, respectively; additional nucleotides inserted during recombination are not pictured). Note that the constant chain segment is separated from the V(D)J portion in the DNA and unspliced mRNA, but contiguous in the mature mRNA. Right: Broad characterization of approaches used for BCR sequencing are shown here for mRNA capture. Bulk approaches focus primarily on the deep sequencing of amplified heavy/light chain regions in an unpaired state. Paired-chain sequencing joins these products in a sequestered PCR reaction (e.g., in an emulsion). Single-cell RNA sequencing relies on separate sequencing on heavy and light chains in the presence of a cell identifier (either a cell barcode in emulsions or a well ID for plate-based approaches). Created with BioRender.com.

Our ability to analyze the B cell repertoire has been greatly increased in the last decade by successive developments in sequencing and single-cell technologies (Fig. 2). Because the CDR3 region of the heavy chain is highly diverse, initial approaches have focused on using next-generation sequencing of this specific region (Weinstein et al., 2009), or later of a broader segment of the heavy chain, to define B cell clones. Specifically, primers specific for the constant chain and a degenerate set of primers binding all V regions are used to tag and identify V, D, and J segment usage as well as the sequence of the CDR3 region, and clones are generally defined based on shared segment usage and CDR3. One of the major advantages of this approach is that it can easily be scaled to millions of receptors and therefore sample a robust fraction of the total repertoire. However, the main drawback is that because the information of the corresponding light chain is lost, this method does not allow the direct production and testing of the original antibody. Heavy chain sequencing can be performed from either DNA or RNA, with some differences: capture at the DNA level does not allow the identification of the constant segment, which is separated by an intron, while measurements based on mRNA can be influenced by different abundances in different populations—plasma cells, for example, express antibody mRNAs at much higher levels than B cells. It is also worth mentioning that as receptor sequences are generated and amplified in bulk, several caveats can bias the results obtained, such as the preferential amplification of specific V regions and the introduction of PCR errors that can resemble somatic hypermutation or random nucleotides introduced during VDJ recombination in the CDR3 region. The latter issue can be avoided with the use of unique molecular identifiers and deeper sequencing, which allow the reconstitution of a consensus sequence (Shugay et al., 2014). While this is not the focus of this review, important experimental and computational considerations are involved in evaluating these datasets and defining clonality (Miho et al., 2018), which are detailed in work from the Adaptive Immune Receptor Repertoire community (Truck et al., 2021).

Single-cell approaches have been used to directly obtain paired light and heavy chains from a single B or plasma cell, although their scale is more limited than studies of the heavy chain alone. Early approaches focused on plate-based cloning to amplify the light and heavy chain and clone complete antibodies from a single B cell and have reliably been used to study hundreds of antibodies (Brezinschek et al., 1998; Tiller et al., 2008). The advent of droplet-based single-cell genomics has allowed groups to scale these approaches to thousands or tens of thousands of cells in a single experiment (Goldstein et al., 2019; Stubbington et al., 2017). In that setting, each B cell is encapsulated in a water-in-oil emulsion and lysed. RNAs are captured and tagged with a droplet-specific barcode before reverse transcription and whole transcriptome amplification (Macosko et al., 2015). The resulting material can be used to determine gene expression but also for targeted amplification of the heavy and light chains of the BCR, allowing the reconstruction of both variable regions. These sequences can then be cloned to produce and test the antibodies of interest generated by the paired heavy and light chain, as was done extensively for the identification of SARS-CoV2 neutralizing antibodies within a few months of the start of the pandemic (Cao et al., 2020; Liu et al., 2020; Scheid et al., 2021). Because these approaches carry cell-level identifiers (either a well or barcode) and allow consensus sequence reconstruction, they are less susceptible to the biases mentioned above.

Several computational or experimental approaches have tried to bridge the gap between these methods to increase the throughput of paired chain sequencing. Computationally, T cell studies have shown that it is possible to infer pairing from the cooccurrence of heavy and light chains sequenced in split pools, but the main caveat is that the method cannot detect rare receptors, which are unlikely to occur in multiple pools. Experimentally, emulsion PCR strategies that directly join the light and heavy chain into a single product (DeKosky et al., 2015; Devulapally et al., 2018; McDaniel et al., 2016) and therefore bypass the need for cell barcodes represent a promising approach for very high throughput paired repertoire sequencing, but at the cost of additional transcriptomic information.

These platforms also offer the opportunity to directly evaluate the repertoire against an antigen of interest as opposed to the total repertoire or to examine specific B cell subsets. This can be accomplished with any analysis modality by simply sorting the population to analyze with subset markers or labeled antigens, but it is important to note that single-cell approaches can leverage gene expression, barcoded antibodies, or barcoded antigens (Setliff et al., 2019) to study multiple subsets or specificities in a single reaction and scale these analyses.

The at-scale assignment of specificities to an antibody sequence remains a key focus of efforts in the field, especially as B cell epitopes are much less constrained that T cell peptides, which must be linear and of a specific length to bind to MHC molecules. While antibody production remains a gold standard, frameworks focused on motif definition (Truck et al., 2021), or leveraging general protein folding tools for antibody design (Bennett et al., 2024, *Preprint*) are likely to transform this field in the coming years. Importantly, multiple platforms have been developed for the study of specificities present in the serum through either the use of phage display (Bourgonje et al., 2023; Larman et al., 2011; Xu et al., 2015), yeast display (Klein et al., 2023; Wang et al., 2022), or using DNA-barcoded antigens (Credle et al., 2022). These approaches do not generally enable the identification of the antibody per se but will be a key resource for the identification of immunodominant antigens (from self and non-self) in populations of interest, and these antigens can be used to sort and profile relevant B cells and plasmablasts. Specifically in the context of autoantibodies, phage-display approaches can define specific autoantigens and motifs targeted by antibodies, including, for example, reactivities that emerge before diagnosis (Bodansky et al., 2024; Vazquez et al., 2022; Zamecnik et al., 2024). These highlighted multiple relevant antigens such as BEST4 in the intestine, which can then be used to identify and sequence the reactive B cells. At least theoretically, this could be performed at a large antigenic scale with barcodes, although this will require the profiling of many more cells than single-cell methods can currently accommodate.

## Emergence of self-reactivity and autoantibodies

Antibodies against self-antigens, i.e., autoantibodies, are a hallmark of many autoimmune diseases of varied ontogeny. Their role in disease, however, can vary broadly: some conditions are characterized by specific pathogenic antibodies that perturb the function of their target, for example, anti-desmoglein antibodies in pemphigus (Ding et al., 1999), anti-ADAMTS13 antibodies in thrombotic thrombocytopenic purpura (Tsai and Lian, 1998), or anti-contactin-associated protein-like 2 antibodies in autoimmune neuromyotonia (Comperat et al., 2022). Autoantibodies against a range of targets are hallmarks of systemic lupus erythematosus (SLE) (Lazar and Kahlenberg, 2023) and rheumatoid arthritis (RA) (van Delft and Huizinga, 2020), where the core pathogenic event is assumed to be the deposition of immune complexes, which can lead to tissue damage and disease progression. Multiple classes of autoantibodies recurrently occur across different autoimmune diseases, including rheumatoid factor, initially identified in RA, as a class of autoantibodies, generally of the IgM isotype, that bind the Fc fragment of Ig (Franklin et al., 1957). Similarly, antibodies against nuclear antigens and citrullinated proteins commonly occur in systemic autoimmune diseases (Suurmond and Diamond, 2015), and anti-neutrophil cytoplasmic antibodies (ANCA) are commonly detected in vasculitis and ulcerative colitis, where they can inform diagnosis (Bosch et al., 2006).

This prevalence raises an important conceptual question regarding autoantibodies as an unavoidable problem or a functional feature of the antibody repertoire: autoantibody precursor sequences may be retained simply through imperfect tolerance or to avoid excessively pruning the repertoire, some autoantibodies may be directly beneficial in the clearance of debris and apoptotic cells (discussed in Skevaki and Wesemann, 2023). Interestingly, they can sometimes play immunomodulatory roles by neutralizing cytokines, as evidenced by the protective effect of anti-type I IFN antibodies in AutoImmune REgulator–deficient subjects (Meyer et al., 2016), but the association of the same autoantibodies with severe COVID-19 (Bastard et al., 2020). This raises the possibility that anti-cytokine antibodies durably modify an individual’s immune responsiveness to new challenges.

Autoantibodies can emerge at two steps of the B cell response: during B cell development, through recombination and random nucleotide insertion, and during the GC reaction, through somatic hypermutation. In the first scenario, one would expect to find an increase in the abundance of self-reactive antibodies in the mature naïve repertoire, while the second process would be restricted to GC B cells, memory B cells, or plasma cells and likely tied to the exposure to specific microbes with some homology to host proteins. Broadly speaking, the mechanisms that prevent self-reactive naïve B cells from completing their development or participating in responses (deletion or anergy) (Nemazee, 2017) remain better understood than those involved in preventing the de novo emergence of self-reactivity in the GC (Brink and Phan, 2018), and here, we will primarily focus on the repertoire-level analysis of these processes in different autoimmune diseases.

## Developmental tolerance pathways in the bone marrow and the periphery

Reactivity to self is common in newly rearranged BCRs (Wardemann et al., 2003) and is associated with longer CDR3 regions and positively charged amino acid usage. These self-reactive B cells are normally depleted from the mature B cell pool at two independent checkpoints, one in the bone marrow and one in the spleen. In the bone marrow, up to 90% of the IgM^+^ immature B cells are depleted before reaching circulation (Loder et al., 1999). This occurs either through apoptosis and clonal deletion or by receptor editing (Gay et al., 1993; Prak and Weigert, 1995; Tiegs et al., 1993), where a developing B cell will re-express RAG to continue light chain recombination and potentially produce a non-self-reactive BCR. Importantly, this process can leave a distinct signature on the repertoire as it favors the usage of more distal light chain V segments. Immature B cells that pass this first checkpoint subsequently migrate to the spleen, where they become transitional B cells and undergo further selection before 1–3% of the initial bone marrow pool emerges as mature B cells (Loder et al., 1999). A fraction of these cells, initially identified as IgM^−^IgD^+^, retain autoreactivity but is anergic and hyporesponsive to further stimulation (Duty et al., 2009). This anergy appears to depend on elevated phosphatase and tensin homolog expression and follows a gradient (Smith et al., 2019), potentially coupling increasing autoreactivity with decreasing responsiveness without fully purging the repertoire of these specificities.

The relative contribution of these different checkpoints and mechanisms to tolerance in the naïve repertoire—and the mechanisms involved in their failure in disease—remain difficult to fully assess in humans, but studies of the repertoire have provided important insights. SLE is an autoimmune disease characterized by the presence of autoreactive antibodies against nuclear antigens, phospholipids, and other targets (Lazar and Kahlenberg, 2023). The breadth of these antibodies has prompted the investigation of defects in early central or peripheral B cell tolerance, and studies of SLE have demonstrated that this process is indeed defective in patients, leading to the retention of 25–50% self-reactive cells in these subjects, compared with 5–20% in healthy individuals (Yurasov et al., 2005, 2006), and similar retention of cells with self-reactivity was described in RA (Samuels et al., 2005). Whether this defect occurs in the bone marrow or splenic checkpoint may depend on antigens, as the presence of anti-HepG2 reactivity was not significantly different in newly emigrant B cells from controls, but anti-cyclic citrullinated peptides (CCP) were absent from this population in controls, while they are found in disease. Overall, the evidence suggests that at least some self-reactivities are enriched in newly emigrant B cells in the context of autoimmune susceptibility (Meffre and O’Connor, 2019), including in healthy subjects carrying the *PTPN22* R620W autoimmune risk variant (Menard et al., 2011). In spite of this, repertoire-level evidence for broadly impaired receptor editing in humans—a process that would primarily occur in the marrow—appears less clear, with some groups reporting increased (Dorner et al., 1998) or decreased (Panigrahi et al., 2008) editing. The repertoires of myasthenia gravis (MG) patients, where disease is driven by autoantibodies against the acetylcholine receptor or muscle-specific kinase, suggest diminished editing based on a lower distance between the recombined V-J segments (Vander Heiden et al., 2017).

Despite the overall reduction in self-reactivity at the splenic checkpoint, many studies in animal models have shown that low-level self-reactivity can be positively selected at this step (Gaudin et al., 2004; Levine et al., 2000). A recent study of humanized mice showed that this process occurs in human B cells but is followed by a regulatory T cell– and MHC class II–dependent negative selection process (Chen et al., 2022). These subsequent positive–negative selection events in the spleen may explain the fact that the repertoire does not seem to follow a simple narrowing path from immature to transitional to naïve cells (Martin et al., 2016). The involvement of T cells in shaping the naïve B cell repertoire is also supported by studies of AIRE-deficient subjects, in which tolerogenic pathways in the bone marrow appear functional, but autoreactive mature B cells emerge as similar frequencies as in SLE or RA patients (Sng et al., 2019). Type I IFN signaling also appears to be a potent modulator of splenic selection in SLE, as it enhances the survival of transitional cells and potentially enables the retention of more autoreactive cells in the repertoire (Dieudonne et al., 2019; Liu et al., 2019).

Regardless of the specific checkpoint, differences in developmental tolerance are expected to result in changes in the pre-antigenic repertoire, which can be inferred from the study of sequences that lack somatic hypermutation and are associated with an IgM/IgD isotype. A joint study of multiple autoimmune diseases reported broad differences in the pre-antigenic repertoire of early-disease SLE but also Crohn’s disease (CD), Behcet’s disease, and eosinophilic granulomatosis with polyangiitis (EGPA) in terms of IGHV usage, and these differences were by comparison minor in ANCA or IgA vasculitis (Bashford-Rogers et al., 2019). Many of these differences persisted in the mutated/switched repertoires, and indeed across many studies of gene usage across B cells and diseases have reported shifts in V segment usage (reviewed in Bashford-Rogers et al., 2018; Foreman et al., 2007). Importantly, many of the studies discussed here assessed the repertoire in the absence of a comprehensive sequencing of the germline Ig loci, therefore some of the associations uncovered may be linked to disease-associated germline configurations in addition to selection events.

Among these shifts in V usage, IGHV4-34 is a prototypical example of changes in selection. This segment encodes antibodies with natural self-reactivity that is dependent on a framework region motif (Pascual et al., 1991), but also the ability to bind commensal bacteria (Schickel et al., 2017). It is present in the naïve repertoire of healthy subjects but expanded in the naïve repertoire of SLE, EGPA, CD (Bashford-Rogers et al., 2019), and MG (Vander Heiden et al., 2017) patients. Interestingly, this segment is also frequently found in leukemic B cells (Xochelli et al., 2017), which may be consistent with an increased tonic signaling in cells that express this BCR. The expansion of this family is therefore consistent with a defective elimination of autoreactive B cells from the naïve repertoire in multiple autoimmune diseases.

## Selection and diversification during B cell activation

As mentioned above, the Ig genes are modified in two critical ways during B cell activation: somatic hypermutation and class-switch recombination. Both processes are dependent on the activity of AID (Muramatsu et al., 2000), and somatic hypermutation is tied to the dedicated structure of the GC, where B cells undergo rounds of mutation and competition for antigen and T cell help, ultimately producing high-affinity antibodies (Victora and Nussenzweig, 2022). While the high expression of AID in GC cells has long suggested that class-switch recombination occurs in GCs, current evidence supports a model where switching primarily occurs outside of the GC (Roco et al., 2019). In addition to changes to the antibody’s specificity and function, the outcome of the GC reaction will also establish B cell fate, broadly speaking either to a memory B cell phenotype or to an antibody-secreting plasma cell (Akkaya et al., 2020). While this is considered the canonical path of B cell activation and differentiation, it is important to note that B cells can be activated outside of GCs through extrafollicular responses (Elsner and Shlomchik, 2020). These extrafollicular structures occur more frequently in autoimmune diseases such as lupus and may contribute to pathogenic antibodies through short-lived plasma cells, while long-lived plasma cells derive primarily from GC-matured B cells. Even though somatic hypermutation was long considered a hallmark of the GC, there is evidence that it occurs in extrafollicular responses (Schroder et al., 1996; Stott et al., 1998), but studies of this pathway in humans still await more confirmation through, for example, in situ repertoire studies.

### Somatic hypermutation and self-pathogen mimicry

Somatic hypermutation presents a key challenge for the establishment of tolerance, as GCs must and indeed do actively prevent the emergence of newly self-reactive cells (Chan et al., 2012). This process can even lead to the “redemption” of previously self-reactive BCRs such as those derived from IGHV4-34 (Sabouri et al., 2014), but it is also clear, based on the evidence of extensive hypermutation, that many autoantibodies derive from a GC reaction and that this tolerance checkpoint can be bypassed in disease (Brink, 2014; Brink and Phan, 2018). In the context of repertoire sequencing, hypermutation is marked by the presence of related descendants of a naïve B cell, enabling the reconstruction of lineage trees among B cells (Abdollahi et al., 2023; Hoehn et al., 2016). Specific considerations are, however, important both because of the nature of clonal evolution and of the fact that the root of the tree—i.e., the unmutated V(D)J sequence—is partially known but contains both random nucleotides and potentially uncharacterized genetic variation. These approaches are particularly useful in the context of a known specificity, for example, when looking at anti-citrullinated protein antibodies in RA (Tan et al., 2014) or anti-desmoglein antibodies in pemphigus (Qian et al., 2007). At the level of the entire repertoire, it is interesting to note that the diseases with the largest changes in gene usage in the naïve repertoire (SLE, EGPA, and CD) were also the ones with the largest clonal expansion and clonal diversification (Bashford-Rogers et al., 2019). Whether this is coincidental or reveals shared susceptibilities in the splenic and the GC checkpoints remains to be investigated.

One case where GCs may directly facilitate the emergence of self-reactive antibodies is in the context of homology between a pathogen-derived and a human protein, a phenomenon described as molecular mimicry (Rojas et al., 2018). For example, in the case of multiple sclerosis, an autoimmune disease associated with Epstein-Barr virus infection (Ascherio et al., 2001; Bjornevik et al., 2022), studies of the circulating and cerebrospinal repertoires identified monoclonal antibodies specific to both the viral protein EBNA1 and the adhesion molecule GlialCAM (Lanz et al., 2022). The association between pemphigus folaceus and leishmaniasis infections in endemic regions in Brazil (Calonga-Solis et al., 2023) supports a similar mechanism, where an antigen from the sand-fly vector has partial homology to desmoglein (Qian et al., 2012). However, in some cases, the association between the development of autoimmunity and a pathogen could be explained simply by a relaxation of tolerance checkpoints in the absence of direct homology, for example, in the context of high interferon levels. This likely occurs in severe viral infections such as COVID-19, where a broad range of autoantibodies have been identified (Credle et al., 2022; Klein et al., 2023; Wang et al., 2021) and are unlikely to all represent homologies with viral proteins, although understanding the extent of mutations and the longevity of these antibodies will be important to clarify whether they derive from GC reactions or through enhanced extrafollicular responses during severe inflammation.

### Regulation of newly emerging self-reactive B cells

Aside from the case of mimicry, where maturation of pathogen specificity and self specificity goes hand in hand, it is evident that normally functioning GCs select against responses to autoantigens. In a particularly elegant study, Singh et al. (2020) studied the development of pathogenic, cold-aggregating, autoantibodies against Ig and performed both V(D)J sequencing and genomic sequencing of lymphoma-associated genes. They found that mutations that occur in lymphoma and affect signaling (for example, in *CARD11*, *TNFAIP3*, and *KLHL6*) precede pathogenic V(D)J variation. This suggests that these pathogenic autoantibodies can only emerge in the context of altered signaling that enables the survival of the B cells expressing such receptors. Besides B cell–intrinsic evolution, it is also clear that T cells play an essential role in regulating the emergence of B cell self-reactivity, as patients with T cell defects show a high level of autoantibodies, for example, in patients with *FOXP3* mutations (Tsuda et al., 2010).

Follicular helper T cells (Tfh) in particular provide key survival and differentiation signals to GC B cells (Crotty, 2014) and, much like for B cells, multiple central and peripheral tolerance mechanisms are in place to prevent the activation of self-reactive T cells (Xing and Hogquist, 2012). This suggests that B cells diverting away from the acquisition of pathogen-derived antigens will lose access to T cell help. There is also accumulating evidence for a more direct suppression by follicular regulatory T cells (Tfr), initially discovered in human tonsils as FoxP3 expressing cells with Tfh features (Lim et al., 2004) and further characterized through murine studies (reviewed in Stebegg et al., 2018). In humans, these cells appear to derive from either regulatory T cells or Tfh cells (Le Coz et al., 2023), with the former potentially being selected for self-reactivity. In this case, acquisition and presentation of self-antigens by B cells may directly mark them for suppression by Tfr cells, and murine studies indeed suggest that this is relevant to prevent the emergence of antinuclear antibodies (Ke et al., 2023).

### Class-switch recombination

Class-switch recombination is a deletional process that uses AID-generated double-strand beaks to bring a more distal constant chain in proximity to the recombined VDJ segments at the heavy chain locus (Stavnezer et al., 2008). The selection of the isotype appears to be mainly regulated by cytokines derived from helper T cells and other immune cells (reviewed in Moens and Tangye, 2014), but rearrangements to IgG4 and IgE, seem more likely to occur through successive switching than via a direct switch from IgM/IgD (Horns et al., 2016).

As their name indicates, IgG4-related diseases (Stone et al., 2012), including pemphigus, are associated with pathogenic IgG4 antibodies. Interestingly however, the constant chain of IgG4 has weak to negligible binding to both the complement molecule C1q and the Fcγ receptor, suggesting that these antibodies are causing disease solely through variable-region dependent mechanisms, for example, desmoglein antibodies disrupting cadherin binding and junctions in the skin. Interestingly, a switch to IgG4 is also observed in recurrent activation, such as repeated mRNA vaccination (Irrgang et al., 2023), and may be a protective mechanism to limit the inflammation generated by highly matured antibodies. IgA-switched versions of common RA autoantibodies such as rheumatoid factor and anti-CCP also occur and may delineate more severe disease forms (Sieghart et al., 2023; Svard et al., 2011) and could be induced by interactions with specific microbes (Chriswell et al., 2022). In addition to isotype switching, antibody constant chains can be further modified by glycosylations that modulate their function, and these change in multiple autoimmune conditions (reviewed in Seeling et al., 2017; Zhou et al., 2021).

While these changes in isotype or glycosylation are key biomarkers of disease, whether this represents a true disease-inducing process remains unclear at this stage, as, for example, increased IgG4 levels could be expected merely through the presence of sustained GC reactions. Indeed, surveys of B cell repertoires have reported changes in switches that would be consistent with changes in the inflammatory environment (Bashford-Rogers et al., 2019). It is interesting to note however that the RA risk gene *AFF3* has been mechanistically linked to changes in class-switching in murine B cells, suggesting that preferential switching can play a role in some autoimmune diseases (Tsukumo et al., 2022).

## Memory B cells, plasmablasts, and plasma cells

Antibody-secreting cells generated from the B cell pool can be either short-lived plasmablasts, which primarily derive from pre-GC B cells and provide rapid antibody production, or long-lived plasma cells, which derive from post-GC B cells and establish in the bone marrow niche or mucosal tissues (Nutt et al., 2015).

In the context of lupus, the short-lived plasmablast compartment that derives from extrafollicular responses has been shown to contribute significantly to the secretion of autoantibodies, including from unmutated IGHV4-34 clones (Jenks et al., 2018, 2019; Tipton et al., 2015). This differentiation path appears to depend on TLR7, thus linking the development of these cells to a core SLE pathway. Interestingly, short-lived plasmablasts also expand in acute viral infections such as COVID-19, where they can cause autoimmune symptoms (Woodruff et al., 2022). This short-lived pathway is directly relevant to therapeutics, as diseases driven by a continuous generation of short-lived plasma cells should be responsive to B cell depletion through rituximab treatment, as is observed in pemphigus vulgaris (Ahmed et al., 2006; Joly et al., 2007). By contrast, large-scale trials of rituximab in SLE failed to meet clinical endpoints (Merrill et al., 2010; Rovin et al., 2012), suggesting a role for long-lived plasma cells in this and other diseases. These extrafollicular responses also appear associated with the emergence of so-called age-associated B cells, a T-bet-expressing subset of antigen-experienced B cells that is expanded in lupus and other conditions (Jenks et al., 2018; Wang et al., 2018). Indeed a recent study showed that these cells depend on the transcription factor ZEB2, which represses GC fate (Dai et al., 2024) in both mice and *ZEB2* haploinsufficent patients.

As they are both generated through the GC reaction, switched memory B cells and long-lived plasma cells represent distinct functional states within the same lineage. Consistent with this idea, Ig sequences from these two populations share similar characteristics (Ghraichy et al., 2021), but the plasma cell repertoire appears much more focused, while memory B cells maintain a large amount of sequence diversity. This is consistent with the idea that long-lived plasma cells maintain circulating levels of high-affinity antibodies that prevent reinfection while memory B cells provide a diverse pool from which responses to variants can be generated in the case of secondary infection with a related pathogen (Akkaya et al., 2020). The study of autoreactive long-lived plasma cells is, however, hampered by the limited access to the bone marrow niche in patients. Nevertheless, the presence of highly mutated self-reactive antibodies in disease is likely associated with established long-lived plasma cells, which would not be eliminated through rituximab therapy.

In addition to the bone marrow, antibody-secreting cells also establish in the mucosa, especially for the local secretion of IgA, and in inflamed tissues, such as the synovium in RA (Doorenspleet et al., 2014; Elliott et al., 2020). The repertoire analysis of these cells shows an expansion of several clones, including IGHV4-34 antibodies, with some sharing with circulating cells. These features are reminiscent of the characteristics of the extrafollicular response observed in SLE. In the intestine, studies of ulcerative colitis have demonstrated an expansion of IgG-secreting cells (Scheid et al., 2023; Uzzan et al., 2022). Inflammation was associated with a reduced repertoire diversity and an increase in CDR3 length, although there was no evidence of marked polyreactivity. Uzzan et al. (2022) identified an autoantibody specific for the integrin αvβ6, although we did not identify broad autoreactivity in intestinal plasma cells of either healthy or ulcerative colitis subjects, and most antibodies profiled appeared to bind to bacterial antigens. While still limited, these studies of local plasma cell compartments raise an interesting question about the role of tertiary lymphoid tissues (TLO) in the emergence of autoantibodies: while extrafollicular responses bypass the GC checkpoint altogether, one could imagine that the tolerance checkpoint enforced by TLO is weaker than in bona fide GCs. This would be consistent with the emergence of local autoantibodies in cancer-associated TLOs (Sharonov et al., 2020), but this association could also simply derive from the fact that inflammation supports both the development of TLOs and the emergence of autoantibodies.

## Conclusions and future directions

Antibodies generated from recombined germline sequences enable the recognition of an almost unlimited set of antigens, and the development of next-generation sequencing and single-cell approaches is now enabling the assessment of these repertoires at scale. Specifically in the context of autoimmunity, studies of repertoires have demonstrated that multiple independent checkpoints are involved in the prevention of self-recognition. This occurs before antigen encounter in both the bone marrow and the spleen, where the secondary process is at least partly enforced by T cells, suggesting a complex interplay between B and T cell tolerance. Upon antigen exposure, at least two independent processes prevent the production of autoantibodies by restricting extrafollicular generation of self-reactive plasma cells and limiting the emergence of de novo autoreactivity in the GC, a process that again involves crosstalk with the T cell repertoire. These different checkpoints are particularly relevant to therapeutic approaches, as rituximab efficiently targets B cells and the replenishment of short-lived plasma cells, but does not affect the long-lived plasma cell compartment.

Importantly, much of our understanding of the mechanisms that occur within the bone marrow or GCs is derived from animal models where these compartments are readily accessible, and repertoire studies of bone marrow samples, lymph node aspirates, or tonsil organoids, among others, will be essential to refine our knowledge of these processes in humans. With these tools in place, deeper studies of the B cell repertoires across different cell states, tissues, and diseases offer new opportunities to identify disease-modulating antibodies and to design specific therapeutic approaches, as it is evident that distinct autoimmune diseases represent failures of tolerance at different checkpoints. Further technical and computational developments, particularly to associate antibody sequences to specificity at a larger scale, will also be essential to enable these studies and refine our understanding of immune tolerance.
